# Major Orthopaedic Surgery in Persons with Haemophilia A with and without Inhibitors Treated by Emicizumab: A Mid-Term, Large, and Successful Series at a Single Center

**DOI:** 10.3390/jcm13092646

**Published:** 2024-04-30

**Authors:** Christian Carulli, Giovanna Daniele, Silvia Linari, Lisa Pieri, Mariastefania Littera, Matteo Mazzetti, Carlo Tamburini, Domenico Prisco, Giancarlo Castaman

**Affiliations:** 1 Orthopaedic Clinic, University of Florence, 50121 Florence, Italy; giovanna.daniele10@gmail.com; 2 Center for Bleeding Disorders, Careggi University Hospital, 50139 Florence, Italy; linaris@aou-careggi.toscana.it (S.L.); castaman@aou-careggi.toscana.it (G.C.); 3 Interdisciplinary Internal Medicine, Careggi University Hospital, 50139 Florence, Italy

**Keywords:** haemophilia, synovitis, arthropathy, inhibitors, knee arthroplasty, hip arthroplasty, amputation, revision, infection, loosening, oxidized zirconium

## Abstract

**Introduction:** Patients with Haemophilia (PWH) need orthopaedic treatments and often they undergo surgery. Classically, PWH with inhibitors have to face such procedures earlier than other patients. Major orthopaedic surgery is not easy and complications are frequent. Emicizumab is the first monoclonal antibody introduced for haematological prophylaxis for PWH with inhibitors, achieving an efficacious haemostasis also in patients with severe haemophilia A with inhibitors, later demonstrated for PWH without inhibitors. A few years ago, emicizumab was also proposed for PWH undergoing surgery, as it supports excellent bleeding control. The literature on orthopaedic surgery using an emicizumab protocol is scarce: only isolated case reports with short-term follow-ups are available. **Aim:** The purpose of this study is the assessment of the mid-term outcomes of major orthopaedic surgery performed in a population of patients with and without inhibitors and an emicizumab regimen. **Methods:** We reviewed the records of 13 PWH (eight with high-titre inhibitors, five without) with a mean age of 54.6 years, undergoing 15 orthopaedic surgical procedures between 2017 and 2022: primary knee and hip arthroplasty, revision, pseudotumor excision, or amputation. Their prophylaxis consisted of the combination of emicizumab and boluses of rFVIIa (PWH with inhibitors) or rFVIII (PWH without inhibitors). The clinical parameters of evaluation were: VAS, Haemophilic Joint Health Score (HJHS), and standard radiologic studies. Follow-up was conducted at 1, 3, 6 months, and then yearly. The survival rate of all implants was also assessed. **Results:** The mean follow-up was 38.8 months (range: 12–65). All patients were successfully treated without complications during surgery. During the postoperative period, a patient affected by a septic complication two months after his pseudotumor excision underwent an above-the-knee amputation. All patients were regularly discharged to the rehabilitative ward, reporting satisfaction for pain reduction and improved joint and global function at the VAS and HJHS scores. No revisions or implant failures were recorded. **Conclusions:** A prophylaxis regimen with emicizumab and factor replacement in PWH with or without inhibitors undergoing major orthopaedic surgery ensures effective bleeding control and good postoperative clinical outcomes at mid-term follow-up, and may be routinely adopted in dedicated high-volume hospitals. This series is the most consistent to date reported at a single Haemophilia centre.

## 1. Introduction

Haemophilia is one of the most frequent rare diseases, consisting of an X-linked bleeding disorder due to the deficiency of coagulation factors VIII (haemophilia A) and IX (haemophilia B), associated with high morbidity and mortality without specific treatment. Patients with the severe forms (FVIII/FIX < 1 U/dL) may suffer from recurrent joint bleeding, muscle hematomas, and an increased risk of life-threatening bleeding, including cerebral haemorrhage. The advent of haematological prophylaxis based on periodic administrations of concentrates containing the deficient factor almost two decades ago has led to a marked improvement of the clinical phenotype of the disease [1]. By that time, the main feature of the disease was the tendency to experience recurrent joint haemorrhages, inducing a chronic synovitis and early arthropathy [2]. This peculiar joint pattern is substantially a severe type of secondary arthritis, associated with early onset of pain, functional limitation, and heavy impact on the quality of life since childhood. After the introduction of the modern haematological care (primary prophylaxis), this complication has significantly decreased; it is expected that in the next decade, fewer cases of highly degenerated joints will be present in young PWH, at least in Western countries. Unfortunately, the effects of joint bleeding occurring in PWH that have not taken advantage of the early prophylaxis are still present in many young and adult PWH. In these symptomatic subjects, several treatment options are proposed, depending on the severity of joint alterations. Oral analgesics, physical maintenance, physiotherapy, use of braces, and intra-articular injections with several types of products (hyaluronic acid, rifampicin, radiocolloids) are the best treatment options now available in early or mild stages of arthropathy [3,4,5]. In cases of poor clinical effects or in the worst cases, surgery is needed: need for arthroscopy is indicated by the presence of persistent synovitis and soft tissue alterations with low grades of arthropathy, while joint arthroplasty remains the gold standard for end-stage haemophilic arthropathy [6,7].

Some PWH develop so-called “inhibitors”, or alloantibodies, against the coagulative factors used for treatment (generally after the first infusions) and are more prone to severe bleedings despite factor administration [8,9]. PWH with inhibitors have been considered the most challenging patients to be managed, particularly due to the inefficacy of replacement treatments, their young age, early presentation of symptoms, and high rates of serious complications in case of surgery; it is the reason why very few centres in the world have historically managed such patients [10,11,12,13,14]. In these patients, despite good functional outcomes, surgery has been usually associated with recurrent postoperative bleedings, dramatically high incidence of postoperative infections and septic loosening, and they often need multiple surgeries [13,14]. The prophylactic regimens adopted in the perioperative period of PWH with inhibitors consisted of the use of by-passing agents, such as recombinant activated FVII (rFVIIa) and Activated Prothrombin Complex Concentrates (APCC) [10,11]. Both demonstrated safety and efficacy, with no superiority of one to the other, with rFVIIa being used not only by bolus administrations, but also by continuous infusion [13]. However, complications have been reported. Very few series have been reported so far with an acceptable number of complications [13].

Recently, a new agent has been tested with outstanding outcomes. Emicizumab is a humanized, monoclonal bi-specifc antibody mimicking FVIII function by bridging together FIXa and FX; it has been initially introduced for prophylaxis in patients affected by haemophilia with inhibitors [15]. Dramatic positive effects on bleeding prevention have been shown by this regimen and its more comfortable way of administration (subcutaneous weekly or longer interval injections). These exciting clinical results prompted the approval of emicizumab also for PWH without inhibitors. As a consequence, several patients with HA, both with and without inhibitors, are candidates for surgery and are now on emicizumab and require additional treatment with replacement agents. In these settings, emicizumab has been associated almost exclusively with rFVIIa (in PWH with inhibitors), because of the risk of thrombotic events when APCC is used in patients taking emicizumab at 100 U/Kg/daily for ≥24 h [16] or rFVIII in PWH without inhibitors. Excellent outcomes in terms of bleeding prevention have been reported, even if few centres and isolated case reports with short-term follow-up are available for the scientific community [17,18,19,20]. A single-centre, cumulative, multispecialty, large series of surgical procedures using an emicizumab regimen has been recently reported, with very good clinical results [21]. Surgical management and outcomes in patients the HAVEN1-4 studies, including patients with and without inhibitors, have been recently reported with good outcomes [22], despite not containing further details on orthopaedic procedures. However, very few major orthopaedic interventions have been reported without specific details about short- and mid-term outcomes. Furthermore, very recently the haematological outcomes of patients with inhibitors on emicizumab included in the Stasey study and who were undergoing several surgical procedures, again including a few major orthopaedic interventions, has been reported [23].

The aim of the present study is the assessment of the mid-term orthopedic and functional outcomes of major orthopaedic surgeries performed in a population of PWH with and without inhibitors on emicizumab and treated with replacement therapy, the most consistent study to date reported at a single Haemophilia centre.

## 2. Materials and Methods

### 2.1. Patient Selection

We reviewed the medical records of all PWH with and without inhibitors (inhibitors titre >5.0 Bethesda units/mL) undergoing prophylactic regimens with emicizumab, who were candidates for major orthopaedic surgery, and who were followed at the authors’ institution from 2017 (the year of introduction of emicizumab for experimental purposes) to 2022. The overall population of patients was represented by 13 subjects (15 procedures: 12 for PWH with inhibitors, 3 for PWH without inhibitors). Inclusion criteria were: adult PWH with and without inhibitors on prophylaxis with emicizumab; candidates for major orthopaedic procedures (primary joint replacement, revision arthroplasty, pseudotumor excision, or limb amputation); minimum follow-up of 12 months. The Institutional Review Board approved the study, and all patients were informed and gave consent at the time of surgery about the treatment, the characteristics of the study, and the need for follow-up.

The mean age of patients at the time of surgery was 54.6 years (range: 32–66); 8 patients had haemophilia A with inhibitors, while 5 had haemophilia without inhibitors. In 12 cases, a previous history of hepatitis C was present, resolved by immunotherapy years before surgery in 11 subjects; 1 patient presented with HIV infection, in good balance with anti-retroviral treatment and periodic clinical follow-up. Nine patients out of 11 had previous orthopaedic surgery before the last surgery, all of which involved multiple joints. All demographics and preoperative clinical data are reported in Table S1.

### 2.2. Surgical Procedures

All surgeries were conducted using general anaesthesia, antibiotic short-term prophylaxis (preoperative infusion of vancomicin 1 g + cephazoline 2 g, repeated 24 h after surgery), and a tailored bleeding prevention protocol.

Total Knee Arthroplasties (TKA) were performed by a standard longitudinal parapatellar approach, application of a tourniquet, and using Legion^®^ Posterior Stabilized or Constrained implants, with oxidized zirconium femoral components (Smith & Nephew, Memphis, TN, USA); no drains were used. The choice of oxidized zirconium components was made due to their demonstrated tribological properties in PWH: two previous patients’series showed mid- and long-term survival rates of this biomaterial in young high-demand patients with a life-long expectancy [24,25].

All Total Hip Arthroplasties were performed by a modified lateral approach, using G7^®^/Taperloc Microplasty^®^ cementless implants, with ceramic femoral heads (Zimmer-Biomet, Warsaw, IN, USA). Also, in these cases due to the young age of PWH, as shown in the past, highly technological implants with highly osteoconductive coatings and ceramic-on-polyethylene couplings were adopted [26,27].

TKA revisions (rTKA) were made using Legion Revision^®^ implants (Smith & Nephew, Memphis, TN, USA) with oxidized zirconium femoral components, cementless stems, and cemented wedges depending on the bone loss or ligament incompetency.

THA revisions (rTHA) were performed using customized acetabular cages coupled with dual mobility cups (in one case a Link^®^ system, Waldemar Link Gmbh, Hamburg, Germany; in another, Lima^®^, San Daniele del Friuli, Udine, Italy) on the pelvic side due to huge bone loss (in both cases two previous revisions were already made). On the femoral side, cemented megaprostheses were used (Link^®^ Mega C, Waldemar Link Gmbh, Hamburg, Germany). No drains were used.

Excisions of any pseudotumors were managed by debridement and the use of heterologous bone chips from the local tissue bank; finally, amputations were both above-the-knee. Of note, one amputation was made in a patient who had previously undergone a pseudotumor excision, and another in a patient who underwent a TKA and an amputation on the contralateral leg due to deep infection and full leg mortification after enteritis-induced sepsis (Table S2).

### 2.3. Haematological Prophylaxis

From the haematological point of view, all patients were on treatment with a weekly subcutaneous administration of emicizumab (1.5 mg/kg). All patients with inhibitors were treated with 2–3 boluses of 90 µg/kg rFVIIa (Novoseven^®^, Novo Nordisk, Denmark) every 3 h at the beginning of surgery until wound suturing, followed by FVIIa 90 µg/kg every 4 hrs during the first 2 days, every 6 h on days 3–4, every 8 h on days 5–7, twice a day on days 8–14, and daily (including before the rehabilitation, when required) on days 15–20, after which rFVIIa was discontinued. Adjunctive intravenous tranexamic acid (TA) 1 g every 12 h for 7 days was administered. Monitoring of prothrombotic markers (peripheral blood smear for schistocyte detection, platelet count, LDH) was also carried out daily for the first 10 days. All major surgeries in patients without inhibitors were managed under FVIII coverage, with factor activity levels ≥ 50 IU/dL and preferably around 80–100 IU/dL, which were maintained for 7–14 days. Adjunctive intravenous TA 1 g every 12 h for 7 days was administered. FVIII activity levels were obtained using a bovine chromogenic assay for measurement of FVIII replacement levels [28]. Mechanical DVT prophylaxis was provided in all cases and no lower extremity venous Doppler ultrasonography was performed.

### 2.4. Clinical and Radiological Evaluation

All patients were followed at our Haemophilia centre, involving a dedicated multidisciplinary team including haematologists, orthopaedic surgeons, anaesthesiologists, physiatrists, internal medicine specialists, physical therapists, and nurses. Patients were clinically evaluated for range of motion (ROM) and by Visual Analogic Scale, Haemophilia Joint Health Score (HJHS) [29], radiologic Petterson’s score [30], and Magnetic Resonance Imaging (MRI) when indicated. Patients undergoing revision arthroplasty were also evaluated by standard radiology, Computerized Tomography (CT), and periodic preoperative and postoperative blood examinations to ascertain the presence of any septic condition (C reactive protein-CRP, Erythrocyte Sedimentation rate–ESR, blood leucocyte count/neutrophil granulocytes percentage). Finally, in one case of hip arthroplasty failure, a joint aspiration for both bacterial culture and assessment of synovial with blood cell count, synovial neutrophil percentage, and leukocyte esterase were performed.

Clinical and radiographic parameters were evaluated preoperatively and after surgery at specific time intervals. Blood and bone loss, use of grafts or devices, type of implant and fixation, time of surgery, and time of tourniquet were recorded. All complications were evaluated, specifically bleedings, defined as unexpected or prolonged blood losses causing haemodynamic instability (reduction of haemoglobin level of 20 g/L^−1^–1.24 mmol/L^−1^): in any case of further reduction of the haemoglobin level, red cell transfusions were provided and recorded. Osteolysis or radiolucency and the presence of periarticular ossifications were also evaluated at every radiologic study after surgery. After surgery, the stay in the orthopaedic ward was continued for a week: all patients were then discharged to the internal medicine ward in the same hospital, continuing their rehabilitative recovery, for at least two additional weeks, in order to complete the postoperative observation, prophylaxis set-up, pain controlling therapy, haemoglobin blood level monitoring, and for comorbidity assessment.

Finally, the degree of satisfaction for the latest surgery was asked of each patient already operated on in the past with the classic regimen, in order to evaluate any different subjective perceptions.

### 2.5. Statistical Analysis

Statistical analysis was performed using SPSS^®^ statistics software version n° 24 (IBM^®^, Armonk, New York, USA). The non-parametric Kaplan–Meyer estimator was used to assess the survival rate of all primary implants, considering aseptic or septic loosening requiring revision as the endpoint. A subjective analysis to assess the outcomes of subjective evaluation was performed using Fisher’s exact test to allow the comparison of cohorts with small sample sizes and to avoid inadequate approximation, taking *p*-values of less than 0.05 as statistically significant with a 95% confidence interval.

## 3. Results

All patients were successfully treated without any complications during or immediately after surgery. No patient was lost at follow-up, which reached a mean time of 38.8 months (range: 12–65). An effective bleeding control was confirmed during surgery and no adverse events related to the haematological prophylaxis were recorded, either in PWH with or without inhibitors. Blood transfusions were required, as expected for two PWH undergoing THA, and one of these two also underwent thigh amputation, due to a postoperative blood loss > 30%. All patients were monitored for at least seven days (considered the minimum period of postoperative observation and early rehabilitation) and then regularly referred to the internal medicine ward to continue the postoperative monitoring and the rehabilitative period; the mean hospital stay was 17.8 days (range: 14–34). VAS, HJHS, and functional ability of the operated joint improved with a statistical significance (*p* < 0.005), except for the patients who underwent amputation who needed more time and other medical steps for the preparation of the external prosthesis (Table S2). No implant showed osteolysis, radiolucency, or any bone alterations. The Kaplan–Meyer curve showed a 100% survival rate of the implants at the latest follow-up (10 implants out of 10). A single patient, affected by a large pseudotumor in his thigh needed a further surgical procedure, with the same prophylaxis and without intra- or postoperative complications (above-the-knee amputation), due to an infection of the surgical site two months after the index procedure (pseudotumor excision).

Even if not fully quantifiable and judgeable, every patient who was already operated on in the past with a standard prophylaxis, reported a superior satisfaction with the latest surgery in the new regimen for two specific aspects: their faster recovery of weight-bearing in the first postoperative days and gait ability during the whole rehabilitative period, and the positive reaction of the other joints (either the target or “healthy” joints) in the first part of the rehabilitation protocol.

## 4. Discussion

In the present study, we reported successful major orthopaedic surgeries in PWH with and without inhibitors using a prophylactic regimen with emicizumab combined with rFVIIa and rFVIII respectively, with a mean follow-up of more than three years. To the best of our knowledge, this is the first consistent series evaluating orthopaedic outcomes at a single centre with a mid-term evaluation. The safety and efficacy of the haematological management, associated with a specific expertise of our Haemophilia team in a multispecialty dedicated facility, have been achieved with a low rate and severity of complications with respect to other smaller series (Table S3). Moreover, considering the complexity of the perioperative management of PWH with inhibitors undergoing major surgery, outcomes and complications in the present series did not substantially differ from other large series with mid- to long-term follow-up conducted on PWH with inhibitors using a standard prophylactic regimen with rFVIIa and APCC adopted for decades in the past [12,13,31]. It seems that this brand-new haematological regimen may be suggested with an expected non-inferiority. With respect to such series, there is a new specific aspect arising from the subjective impressions of PWH that experienced other major orthopaedic surgeries in the past, related to the recovery of ability of the operated joint and the efficacious response of the other joints during the rehabilitative period. It is surely not quantifiable and scientifically valuable, but there seems to be a typical report of satisfaction of all patients who have been included since 2017 in clinical trials with emicizumab prophylaxis, as well as the experiences of patients with inhibitors after emicizumab prophylaxis went on the market in Italy in 2019, and then in 2020 for those without inhibitors.

Furthermore, even at mid-term follow-ups, the orthopaedic complication rate of the present series is significantly lower than all the other reports available in the literature when dealing with primary and revision arthroplasty [12]. The absence of postoperative bleedings and infections is of paramount importance, even in patients affected by HIV, who in the past have classically experienced several critical clinical issues in several reports [13,14].

Three papers have been recently published with anecdotal, single-case reports, with no complications or a single haemorrhagic event which was quickly solved [16,17,18].

Seaman and Ragni in their report in 2019 described the use of emicizumab in a 54-year-old man with moderate haemophilia A and high-titre inhibitors undergoing THA [17]: the patient had no postoperative bleedings, no blood transfusions were needed, and he reported a substantial improvement in his daily life activity.

Evans and colleagues reported the case of a 48-year-old man with severe haemophilia A and high-titre inhibitors undergoing a two-stage rTKA after an infected TKA, with a regimen of emicizumab and rFVIIa [18]. On the first postoperative day, immediately after physical therapy, he developed a bleeding managed by increasing bolus doses of rFVIIa.

Guillame et al. reported their case of a 44-year-old man with severe haemophilia A without inhibitors undergoing an elbow arthroplasty [19]. The prophylaxis was made by emicizumab and FVIII: no complications were recorded.

Recently, haematological outcomes in patients enrolled in HAVEN 1-4 and Stasey studies have been reported. While the haematological outcome was, in general, good with a wide heterogeneity of haematological prophylaxis for major surgeries, a few major orthopaedic surgeries have been included again with heterogeneous haematological approaches. Furthermore, importantly specific orthopaedic management and functional outcomes have not been detailed [22,23].

Rener et al. reported their huge series with eight major orthopaedic procedures at a follow-up of about three years [20]. Several complications were recorded, mostly severe, as a postsurgical haemorrhagic event after internal fixation for a polytrauma with bilateral fracture of the distal legs, followed by a below-the-knee amputation; a case of non-union after a femoral fracture with a following re-intervention; a case of a septic complication after an ankle arthrodesis and following below-the-knee amputation; and a case of septic complication after TKA. However, five surgeries were performed in an emergency setting and should be considered as potentially life-threatening issues, although no mortality was observed.

Castaman et al. reported a large series of major surgeries performed in PWH with inhibitors using a regimen with emicizumab and rFVIIa with a short- to mid-term follow-up [21]: among these procedures, 10 were orthopaedic surgeries. Three patients needed blood transfusions (two PWH undergoing rTHA and one treated by thigh amputation). Compared to the historical use of rFVIIa for similar surgeries [14], a 40% reduction in its use was observed. A successful rehabilitative period was carried without complications and with high satisfaction of patients.

Regarding the five PWH without inhibitors, there was no need for blood transfusion or any significant intra- or postoperative complications. No differences were substantially perceived or referred by these patients with respect to previous surgical procedures.

This study has some limitations, mainly related to the small group of patients and the mid-term follow-up time. However, as mentioned, it represents the largest cohort of patients uniformly managed from a haematological point of view and with the longest orthopaedic follow-up available to date with this novel combination of continuous prophylaxis with non-replacement therapy and the use of clotting concentrates, with a lower rate of complications with respect to the case reports or limited study population now available.

## 5. Conclusions

A prophylaxis regimen including emicizumab and factor replacement in PWH undergoing major surgery ensures effective bleeding control and good postoperative clinical outcomes at mid-term follow-up, and may be routinely adopted at dedicated centres. In our series, PWH with or without inhibitors have been successfully managed without bleeding issues, and adequate functional orthopaedic recovery recorded at a mid-term follow-up. We are now enrolling more PWH with or without inhibitors to confirm these very good results.

## Data Availability

Further data are unavailable due to privacy or ethical restrictions.

## Supplementary Materials

The following supporting information can be downloaded at: https://www.mdpi.com/article/10.3390/jcm13092646/s1, Table S1: Demographic patient’s data; Table S2: Surgical and clinical data; Table S3: Comparative results of specific series.
