# Supplementary material for: Major Orthopaedic Surgery in Persons with Haemophilia A with and without Inhibitors Treated by Emicizumab: A Mid-Term, Large, and Successful Series at a Single Center
Source: J Clin Med. 2024 Apr 30;13(9):2646. doi: 10.3390/jcm13092646 (PMC11084841; doi:10.3390/jcm13092646)
Supplement: Supplementary file 1 [file jcm-13-02646-s001.zip › jcm-2887226-supplementary.pdf]

Table S1: Demographic patient's data

**Number of patients**	13
**Age** (mean/range)	54.6 years (32-66)
**Type of Haemophilia**	
**A severe**	12
**A moderate**	1
**Haemophilia Joint Health Score** (mean/range)	Pre-op: 18.4 (16-22)
**Inhibitors**	8/13 patients
**HCV infections**	12/13 patients (11 neutralized)
**HIV infections**	1/13 patients
**Previous surgery**	9/13 patients
**Other target joints**	13/13 patients

Table S2: Surgical and clinical data

TYPE OF SURGERY	N° of cases	Complications	Transfusions	Mean VAS Pre/post-op	Mean HJHS Pre/post-op	Satisfaction
**Total Knee Arthroplasty**	5	-	-	8.3 / 1.1	19.5 / 3.0	Full
**Total Hip Arthroplasty**	2	-	-	9.1 / 0.8	21.2 / 2.5	Full
**Revision Knee Arthroplasty**	2	-	-	7.5 / 1.3	18.7 / 3.6	Full
**Revision Hip Arthroplasty**	3	-	2	7.1 / 1.0	19.0 / 3.1	Full
**Pseudotumor excision**	2	1 (recurrence of infection)	-	6.4 / 2.4	16.6 / 5.3	Full (1) / Partial (1)
**Amputation**	1	-	-	7 / 3	22.0 / 10.4	-

Table S3: Comparative results of specific series

Authors & publication year	N° of major orthopaedic surgeries	Type of Haemophilia & Haematological prophylaxis	Follow-up	Complications
**Seaman and Ragni, 2019**	1 Total Hip Arthroplasty	Mild Haemophilia A and high-titre inhibitors<br><br>Emicizumab & rFVIIa	2 weeks	None
**Evans et al, 2020**	1 Revision Total Knee Arthroplasty (after an infected TKA)	Severe Haemophilia A and high-titre inhibitors<br><br>Emicizumab & rFVIIa	1 month	Bleeding during the first day of post-operative rehabilitation
**Guillaume et al, 2021**	1 Total Elbow Arthroplasty	Severe Haemophilia A<br><br>Emicizumab & rFVIII	5 days	None
**Rener et al, 2023**	5 patients (8 procedures):<br><br>- 4 fixations (1 bilateral)<br>- 1 Total Knee Arthroplasty<br>- 2 below-the-knee amputations<br>- 1 necrectomy after new ankle fusion	Severe Haemophilia A (4 cases)<br>Severe Haemophilia A and high-titre inhibitors (1 cases)<br><br>Emicizumab & rFVIII (no inh)<br>or<br>Emicizumab & rFVIIa (inh)	About 3 years	• 1 severe bleeding in the third day of post-operative rehabilitation needing further surgery (finally, a below-the-knee amputation)<br>• 1 infection (Total Knee Arthroplasty)<br>• 1 infection after ankle fusion (finally, a below-the-knee amputation)<br>• Futher surgery after a femoral fracture non-union
**Present study**	13 patients (15 procedures):<br><br>- 5 Total Knee Arthroplasties<br>- 2 Total Hip Arthroplasties<br>- 2 revision TKAs<br>- 3 revision THAs<br>- 2 pseudotumor excisions<br>- 1 above-the-knee amputation	Severe Haemophilia A (cases)<br>Severe Haemophilia A and high-titre inhibitors (x cases)<br><br>Emicizumab & rFVIII (no inh)<br><br>Emicizumab & rFVIIa (inh)	More than 3 years	3 blood transfusions:<br><br>• 2 after 2 revision THA<br>• 1 after an above-the-knee amputation
